# Predicting late radiation-associated neurocognitive and endocrine toxicity in patients with brain tumors

**DOI:** 10.1007/s11060-026-05646-9

**Published:** 2026-05-28

**Authors:** Kyle Tuohy, Aarav Badani, Ava Franklin, Brad Zacharia, Dawit Aregawi, Alireza Mansouri, Paul Brown, Michael Glantz

**Affiliations:** 1 Department of Neurosurgery, Penn State Hershey Medical Center, Hershey, PA USA; 2 University of California Berkeley, Berkeley, CA USA; 3 University of Virginia, Charlottesville, VA USA; 4 Department of Oncology, Penn State Cancer Institute, Hershey, PA USA; 5 Department of Radiation Oncology, Mayo Clinic, Rochester, MN USA; 6 Department of Neurosurgery – EC110, Penn State College of Medicine, Hershey Medical Center, 30 Hope Drive, Hershey, PA 17033 USA

**Keywords:** Brain tumors, Cognitive/endocrine dysfunction, Cranial irradiation, Machine learning, Neurotoxicity

## Abstract

**Purpose:**

Neurocognitive and endocrine dysfunction are potential complications of cranial irradiation. However, risk factors are poorly understood, impeding accurate prognostication and exploration of potential preventive interventions. The objective of this study was to evaluate the prognostic value of various vascular and genotypic risk factors for the development of radiation-related toxicities.

**Methods:**

This single-institution retrospective cohort study included patients with metastatic and malignant primary brain tumors who received cranial irradiation as part of their initial tumor-directed therapy. Demographic and treatment characteristics were collected, as well as putative vascular and genotypic risk factors. Univariate, multivariate, and machine-learning analyses were performed using five pre-specified measures of radiation-related toxicity. The primary outcome was change in mini-mental status exam (MMSE).

**Results:**

Eighty patients (53% male, mean age 55.7, 53% primary and 44% metastatic brain tumors) were included. Elevated homocysteine and *ApOE4* genotype were the strongest predictors of MMSE decline in the multivariate model (OR 3.96 [6.5–200, *p* < 0.001 and 2.85 [1.92–27.6], *p* = 0.004). Elevated homocysteine was associated white matter change on MRI and both physician and patient assessment. *ApoE4* allele was associated with new endocrine deficiency, and physician assessment. An online nomogram provides risk predictions for each of the five late toxicity outcomes: (https://afranklin22.shinyapps.io/PRMMSERadiationRiskFactors/)

**Conclusion:**

Two pre-treatment laboratory values (elevated homocysteine and *ApOE* genotype) were strongly associated with post-radiation neurocognitive and endocrine dysfunction using a variety of domains. Our predictive algorithm can aid clinicians in stratifying baseline risk and should be validated in prospective trials and with additional metrics of ND.

**Supplementary Information:**

The online version contains supplementary material available at 10.1007/s11060-026-05646-9.

## Introduction

Cranial irradiation remains a mainstay of treatment for primary and metastatic brain tumors, often in conjunction with surgery and chemotherapy. Unfortunately, radiation-induced neurocognitive dysfunction (ND) is a potential consequence of treatment [1–3] and can affect multiple domains of cognitive function, including verbal memory, spatial memory, attention, and problem-solving ability [4–8]. Neuroendocrine dysfunction [9] and post-radiation structural changes on brain imaging [10, 11] have also been well-documented. As a result, the prevention of radiation-induced brain injury is an essential concern in optimizing quality of life in patients with brain tumors [12]. 

The causes of radiation-induced ND are multifactorial, and multiple pathophysiological mechanisms have been implicated, including inflammatory responses caused by oxidative stress, microvascular changes, and impaired function and proliferation of neuronal, glial, and endothelial cells [6]. Vascular injury appears to predominate in viable tissue [6] and may play a critical role radiation necrosis and ND. Radiation-induced vascular changes consist of vessel occlusion, endothelial dysfunction, capillary loss, and perivascular astrocyte hypertrophy, culminating in a breakdown of the blood-brain barrier and edema [13]. Given the similarity in clinical presentation between ND and Alzheimer disease, some investigators have hypothesized that genotypic risk factors for dementia may also predispose patients to ND. The e4 allele of the apolipoprotein 4 (*ApOE*) gene is strongly associated with Alzheimer disease, and patients with brain metastases carrying this allele demonstrated a greater degree of neurocognitive decline after whole-brain radiotherapy (WBRT) [14]. 

Although advances in non-invasive imaging including functional MRI, spectroscopy, and diffusion tensor imaging have facilitated early detection of brain changes in those who develop ND [15], there are no validated biomarkers that can *predict* the risk of ND prior to the start of cranial irradiation. In light of the evidence for vascular injury in patients with ND, and the similarity of ND to other forms of dementia, we hypothesize that the presence of vascular and dementia-related risk factors might identify patients at risk for developing ND following cranial irradiation.

## Methods

### Study design and patient selection

We performed a retrospective cohort study of patients at a single institution who received radiation for intracranial malignancies. This study was approved by the Penn State College of Medicine Institutional Review Board (ID: STUDY0027827). Adult patients (*≥* 18 years) with a primary or metastatic intracranial malignancy who received WBRT or partial brain fractionated, intensity-modulated radiotherapy (IMRT) were eligible for this study. Patients were excluded if they did not receive radiation, were treated with stereotactic or proton beam irradiation, were under the age of 18 at the start of radiation, had a pre-treatment MMSE score of < 27, if any of the baseline predictor variables for this study were missing, if they developed intracranial disease recurrence prior to 6 months, or if they were unavailable for 6-month follow-up.

The pre-specified primary outcome measure, post-radiation cognitive decline, was defined as a mini-mental status exam (MMSE) score of 24 or less at six months following completion of cranial irradiation. Although limitations in the MMSE exist, this remains one of the most widely used tools internationally for the diagnosis and clinical prognosis of cognitive impairment [16, 17]. Additional definitions of post-radiation brain injury included the development of MRI-documented white matter changes at 6 months as determined by the attending neuroradiologist (who was masked to all predictor variables except age), subjective physician assessment and subjective patient assessment of cognitive decline at 6 months, and the development of laboratory-defined endocrine dysfunction at 6 months which was not present prior to the start of cranial irradiation.

The rate of patient and physician assessments of cognitive decline were extracted from visit notes written at the 6-month post-radiation follow-up visit (or at the last clinic visit prior to 6 months in patients with no further follow-up). These reflect patient and physician reported outcomes, but were not formally and prospectively defined. Endocrine dysfunction was assessed biochemically by serum lab values of hypothalamic-pituitary axis hormones below the lower limit of normal.

Pre-specified putative laboratory predictors of post-radiation brain injury including homocysteine, cholesterol, hemoglobin A_1_C, and *ApOE4* genotype were collected prior to the start of cranial irradiation. Demographic (age, gender, tumor type, pre-radiation MMSE), treatment (radiation prescription, concurrent chemotherapy and anticonvulsant treatment), and outcomes data were collected. Temporal lobe irradiation was defined as any radiation plan that include the temporal lobe in the treating field. By definition, all patients who underwent WBRT were included in this group. All patients with grade 4 gliomas received the Stupp protocol including fractionated radiotherapy for a total of 60 Gy in 30 fractions, with concurrent and adjuvant temozolomide. All patients with low grade gliomas received 54 Gy in 40 fractions. Patients with brain metastases received WBRT (30 Gy in 10 fractions). Patients with CNS lymphomas were only treated with radiation at recurrence, and almost entirely received 30 Gy in 12 fractions, but two patients received 45 Gy in 25 fractions.

### Statistical analysis

Descriptive statistics are presented as counts and percentages for categorical data, as means and standard deviations for normally distributed interval data, and as medians and interquartile ranges for non-parametric data. Laboratory values were dichotomized as “normal” or “elevated” when they were within or above the reference laboratory-defined normal ranges. *ApOE4* genotype was dichotomized as “normal” or “abnormal” based on the presence of one or more *ApOE4* alleles. The MM alpha-1 antitrypsin genotype was considered “normal”; all other genotypes were considered “abnormal”. P-values for differences in patient characteristics in the two post-radiation cognitive status groups (MMSE < 24 vs. >24) were obtained using Fisher’s Exact Test for categorical data, and unpaired t-tests for interval data. Multivariate logistic regression was performed to evaluate predictors of brain injury after cranial irradiation for the primary outcome measure (MMSE < 24) and each of the four secondary outcome measures (white matter change, physician assessment of cognitive decline, patient assessment of cognitive decline, endocrine dysfunction). Endpoints were again dichotomized (yes vs. no) with “yes” for the endpoint of white matter change defined as > 3 lesions according to Fazekas [18] and Corn [19]. Characteristics of potential predictive value were selected for the multivariate analysis if the associated p-value in the univariate analysis was *≤* 0.10. For all analyses, a p-value of *≤* 0.05 was considered statistically significant.

A machine learning analysis using both Random Forest [20] and Gradient Boosting [21, 22] classification models was also performed. Training and testing cohorts were generated by randomly splitting patient data into 75% and 25% subsets respectively. The training set was used to train Random Forest and Gradient Boosting models. To ensure robust identification of predictors across different algorithmic implementations, two gradient boosting frameworks were employed: the gradient boost package (v2.1.8) and xgboost (v4.7-2). Random Forest models were trained using: randomForest (., importance = TRUE, ntree = 500) (randomForest R package, v4.7-2). Gradient boosting models were trained using both gradient boost (distribution = “bernoulli”, n.trees = 500, interaction.depth = 3) and xgb.train (…, params = [booster = “gbtree’, objective = ‘binary: logistic, eval_metric = error], nrounds = 100) (xgboost R package v4.7-2). Feature importance was evaluated using Mean Decrease in Accuracy for Random Forest, Relative Influence for gradient boost models (Fig. 2A), and Gain for xgboost models (Fig. 2C). The AUROC performance was evaluated in the test set (performance function, ROCR v1.1-11 R package). For logistic regression classification models the training set was used to train a binomial generalized linear model (GLM) using bayesglm(., family = “binomial”, maxit = 500) (arm R package, v1.12-2). Because of the small size of our patient cohort, we confirmed that model performance was not influenced by choice of partition ratio (50:50, 60:40 and 80:20 train: test splits yielded similar results). The ggplot2 R package (version 3.3.5) was used for data visualization.

The predictive nomogram tool was programmed using RStudio (version 4.5.0), using the Shiny framework, and is hosted on Shinyapps.io, a web-based application. The predictor variables, age, homocysteine level, presence of the ApoE4 allele, and pre-radiation MMSE, were selected based on previously conducted multivariable analyses, identifying which variables held statistically significant relationships with the included outcomes of interest. Each outcome was treated as a binary event, allowing the algorithm to generate the continuous predicted risk estimates. The baseline risk of each outcome was derived from the multivariate model intercepts and compared with reported baseline frequencies from the published literature, which are summarized in Supplemental Table 1. The comparison was not for recalibration or external validation of the model, but for interpretive purposes only. The coefficients from the multivariate analysis were added into the interactive nomogram code, allowing users to input patient-specific variables and generate the continuous predicted risk estimates for said patients. The predicted risk estimates from this application are intended for exploratory purposes only and were not developed or validated for clinical usage.

## Results

**Fig. 1:**
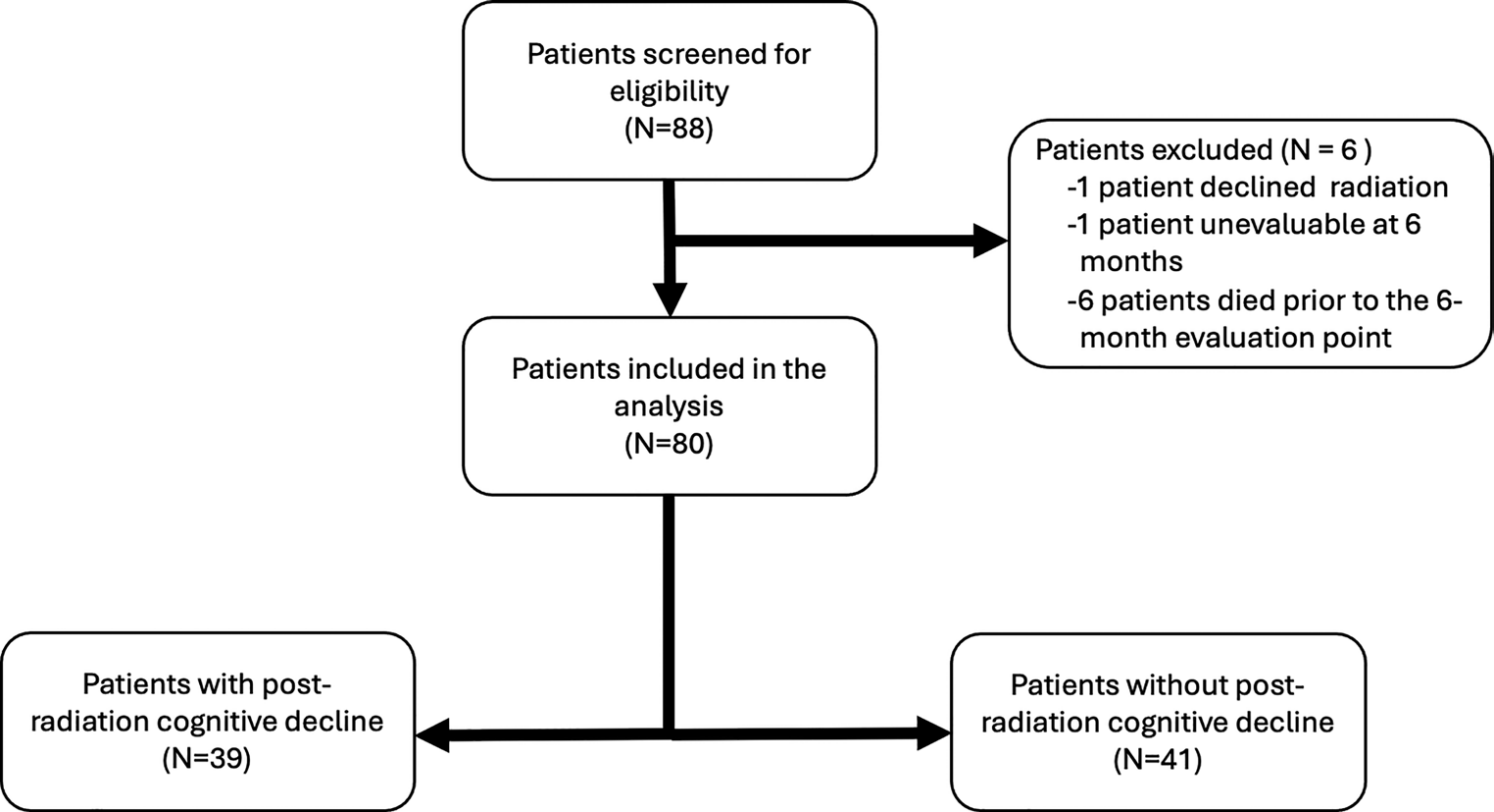
Patient inclusion flow chart

Eighty-eight consecutive patients were eligible for inclusion in this study. One patient declined cranial irradiation, 1 patient was not evaluable at 6 months, and 6 patients died prior to the 6-month assessment point (Fig. 1), leaving 80 patients (91%) to be included in the final analyses. Detailed demographic information for all eligible patients is presented in Table 1. Of these 80 patients, 53% were male and the mean age was 55.7 (SD 12.5). There were 41 patients with gliomas, 7 patients with CNS lymphomas, and 32 patients with brain metastases. In the glioma category there were 30 grade IV gliomas (25 glioblastoma IDH-wildtype and 5 astrocytoma IDH mutant). There were 2 astrocytoma IDH-mutant grade 3 tumors, 2 oligodendroglioma IDH-mutant grade 3 tumors, and 3 oligodendroglioma IDH-mutant grade 2 tumors that represented clinically and radiographically aggressive tumors that were not re-biopsied. There were 4 recurrent meningiomas. One was grade III and three were grade II, however only one of these 4 recurrent tumors (originally classified as grade III) was reoperated. In the CNS lymphoma cohort, there were 4 primary and 3 secondary CNS lymphomas, all recurrent at the time of radiation. In the brain metastasis category, there were 8 patients with non-small cell lung cancer, 5 with small cell lung cancer, 9 with breast cancer, 2 with renal cell carcinoma, 2 with melanoma, 1 each with bladder, colon, prostate, and uterine cancer, and 2 with unknown primary tumors.

**Table 1:** Patient characteristics

Patient Characteristic	All Patients (*N* = 80)	Post-Radiation Cognitive Status		*P*-Value
		MMSE *≥* 24 (*N* = 41)	MMSE < 24 (*N* = 39)	
Age *≥* 65	17 (21%)	4 (9.8%)	13 (34%)	**0.01**
Age (mean, SD, years) (median, range, years)	55.7 (12.5) 56.0 (6–84)	53.6 (13.0) 53 (6–84)	57.9 (11.8) 60 (34–84)	0.13
Male	42 (53%)	24 (59%)	18 (6%)4	0.27
Primary Brain Tumor	45 (56%)	24 (59%)	21 (54%)	0.67
Whole Brain Irradiation	29 (36%)	13 (32%)	16 (41%)	0.39
Temporal Lobe Irradiation	38 (48%)	21 (51%)	17 (44%)	0.49
Concurrent Anticonvulsant Use	32 (40%)	17 (41%)	15 (38%)	0.78
Elevated Homocysteine Level	23 (29%)	2 (1.0%)	21 (54%)	**< 0.0001**
Elevated HgA_1_C Level	10 (13%)	5 (12%)	5 (13%)	0.93
Elevated Cholesterol	50 (63%)	25 (61%)	25 (64%)	0.77
ApoE4 Allele Present	20 (25%)	5 (12%)	15 (38%)	**0.0067**
Pre-Radiation MMSE Score	29.36 (0.82) 30.0 (27–30)	29.46 (0.74) 30.0 (28–30)	29.26 (0.88) 30.0 (27–30)	0.26

The MMSE at 6 months fell below 24 in 39 patients (48.8%). Univariate analysis revealed three factors associated with post-radiation cognitive decline using the primary outcome measure (MMSE score < 24 vs. *≥*24): age *≥* 65 (OR 4.63 [1.36–15.8], *p* = 0.01), elevated homocysteine level (OR 22.8 [4.81–108], *p* < 0.0001), and *ApoE4* allele present (OR 4.5 [1.5–14.0], *p* = 0.0067). No other patient or treatment characteristics, including sex, type of radiation, temporal lobe dose, primary vs. metastatic brain tumor, or concurrent anticonvulsant use was associated with cognitive decline (Table 2).

**Table 2:** Univariate predictors of post-radiation cognitive decline* (*N* = 80)

Potential Predictor	Odds Ratio	95% Confidence Interval	*P*-Value
Age *≥* 65	4.63	1.36–15.8	**0.01**
Whole Brain Irradiation	1.50	0.60–3.75	0.39
Temporal Lobe Irradiation	0.74	0.31–1.78	0.49
Concurrent Anticonvulsant Use	0.88	0.36–2.16	0.78
Elevated Homocysteine Level	22.8	4.81–108	**< 0.0001**
Elevated HgA_1_C Level	1.06	0.28–3.99	0.93
Elevated Cholesterol	1.14	0.46–2.83	0.77
ApoE4 Allele Present	4.50	1.45-14.0	**0.0067**

*Mini-Mental Status Exam Score < 24

Thirteen patients (16.25%) had one or more abnormalities in their hypothalamic/pituitary axis. Overall, 7 patients developed hypothyroidism, 3 developed cortisol deficiency, 2 developed growth hormone deficiency, and 4 testosterone deficiency (16 deficiencies in all). Six patients developed hypothyroidism alone, 2 cortisol deficiency alone, 2 testosterone deficiency alone, 1 testosterone plus cortisol deficiency, 1 growth hormone plus testosterone deficiency, and one growth hormone plus thyroid deficiency. Seven patients also had elevated prolactin levels, but since there were a number of potential confounders (for example, the use of Zofran and other antiemetics in some) we did not count this as a treatment-associated neuroendocrine problem.

Multivariate logistic regression analysis was performed using these three characteristics as covariates for each of the five pre-specified outcome measures (Table 3). Elevated homocysteine was associated with MMSE < 24, white matter change on MRI, and both physician and patient assessment of cognitive decline. *ApoE4* allele was associated with MMSE < 24, new endocrine deficiency, and physician assessment of cognitive decline. Age was not a statistically significant predictor of any outcome measure. Of note, the probability of ‘yes’ under each outcome (except endocrine dysfunction) increased dramatically for homocysteine levels between 9 and 13 when used as a continuous variable (Supplemental Fig. 1). However, reanalysis using age and homocysteine level as continuous variables resulted in nearly identical results.

**Table 3:** Multivariate predictors of neurocognitive dysfunction (*N* = 80)

Outcome Measure	Potential Predictor	Odds Ratio	95% Confidence Interval	*P*-Value
MMSE Score < 24	Age *≥* 65	1.75	0.89–12.2	0.080
	Elevated Homocysteine Level	3.96	6.5–200	**< 0.001**
	ApoE4 Allele Present	2.85	1.91–27.6	**0.004**
New Endocrine Deficiency	Age *≥* 65	0.45	0.06–2.11	0.4
	Elevated Homocysteine Level	1.13	0.25–4.49	0.9
	ApoE4 Allele Present	7.52	2.12–29.5	**0.002**
New White Matter Disease on MRI Scan	Age *≥* 65	0.99	0.56–5.78	0.3
	Elevated Homocysteine Level	3.70	3.30–41.8	**< 0.001**
	ApoE4 Allele Present	1.57	0.80–8.25	0.12
Physician-Assessed Cognitive Decline	Age *≥* 65	0.99	0.53–7.15	0.3
	Elevated Homocysteine Level	4.14	5.65–109	**< 0.001**
	ApoE4 Allele Present	3.23	2.48–35.5	**0.001**
Patient-Assessed Cognitive Decline	Age *≥* 65	0.68	0.47-5.00	0.50
	Elevated Homocysteine Level	3.21	3.28-87.0	**0.001**
	ApoE4 Allele Present	1.83	0.96–11.4	0.068

*Mini-Mental Status Exam Score < 24

**Fig. 2:**
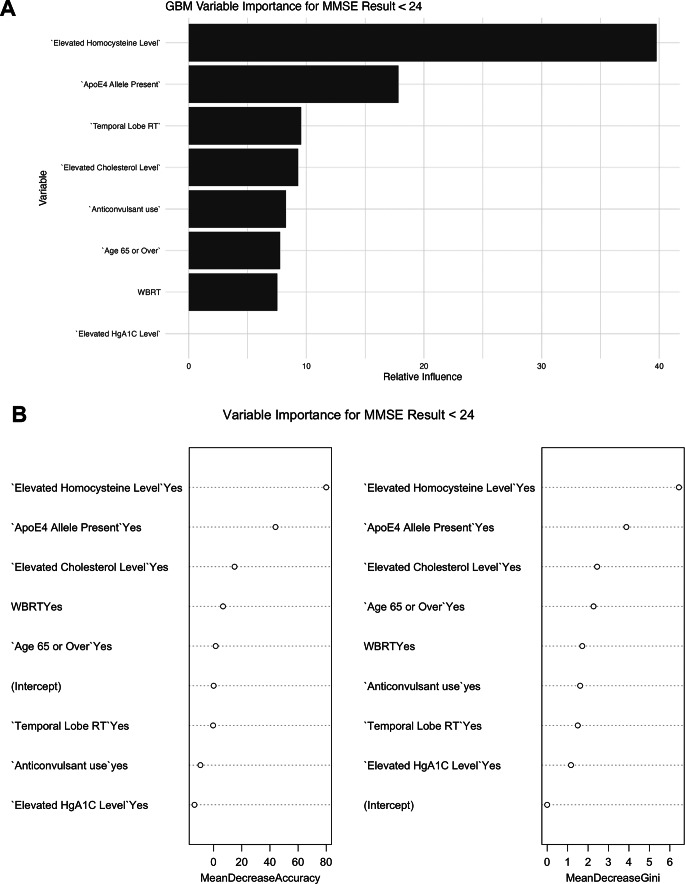
Machine learning analyses. **A**) Gradient Boosting analysis identifying key features with their relative influence on the outcome of MMSE < 24; **B**) Random Forest model presenting variable importance of each risk factor through “Mean decrease accuracy” and “mean decrease gini” with higher values representing greater importance to the outcome of MMSE < 24

Our machine learning analyses corroborated the results of the regression model, highlighting homocysteine level and *ApoE4* as most predictive for post-radiation cognitive decline (Fig. 2). Further gradient boost analyses (XGB) for each of the five outcome measure are presented in Supplemental Fig. 2. A predictive nomogram incorporating these two variables, plus age and pre-radiation MMSE, was developed to produce an estimated risk for each of the five outcomes and is available at https://afranklin22.shinyapps.io/PRMMSERadiationRiskFactors/.

## Discussion

Fractionated external beam cranial irradiation will continue to play a role in the management of patients with intracranial malignancies for the foreseeable future. Because improvements in surgery, radiation, and systemic therapy have extended survival for many of these patients, the late effects of radiation on the brain, and, consequently, on quality of life, are becoming increasingly important considerations to patients and physicians. A variety of strategies, most notably hippocampal avoidance radiation and memantine administration, have been introduced to mitigate this risk [23], and some treatments (for example, donepezil and methylphenidate) may modestly ameliorate the symptoms of radiation-related neurocognitive toxicity, but these interventions are, by necessity, applied to *all* patients undergoing treatment, or only *after* symptoms have developed, because no specific biomarkers are available to predict the risk for ND *before* the start of therapy. Previous studies have identified some patient- and treatment-related characteristics associated with increased risk, including young and advanced age, radiation dose, treatment volume, concurrent chemotherapy, and baseline MMSE score [4–8, 24] and one recent study has proposed an algorithm for prediction of neurocognitive decline based on organ at risk calculations [25], but a simple, quantitative prediction tool based on easily obtained pre-treatment patient characteristics remains elusive. The results of our study suggest that pre-treatment homocysteine level, ApoE4 status, and patient age can accurately estimate the risk of ND (defined in multiple, clinically meaningful ways) *prior* to the start of radiotherapy (Table 3). Based on these findings, we have produced a simple nomogram calculator that can be applied at the bedside to assist in conversations about risk, facilitate shared decision-making, inform the intensity of post-treatment surveillance, and assist with recruitment to and analysis of trials focused on prevention.

In addition to the traditional techniques of logistic regression and decision analysis used to produce Table 3 and our predictive nomogram, we also applied two machine learning analyses (random forest and gradient boost) to our data. Machine learning models do not generate measures of statistical significance or effect size, but are particularly powerful in overcoming confounding and model overfitting that present challenges to traditional statistical techniques, particularly with relatively small sample sizes. Random Forest ranks the relative importance of predictor variables included in a model using an ensemble decision tree approach [20]. Gradient Boost evaluates how much variability in the model can be attributed to each predictor [26]. Both models identified homocysteine level and ApoE4 status as the most critical predictors, corroborating the findings of the multivariate regression analysis. Taken together with conventional inferential statistical approaches, our machine learning models provide a more comprehensive understanding of the factors influencing the development of ND.

The pathophysiology of radiation-induced ND is multifactorial and beyond the scope of this discussion, but has been described in detail elsewhere [27, 28]. Briefly, damage begins hours to days after therapy, with local inflammation, vascular injury, and glial cell dysfunction. In the subsequent months, altered neuronal function and decreased neurogenesis, particularly in the hippocampus, leads to progressive cognitive impairment [28]. The deficits developing after 6 months are considered to be progressive and irreversible, thus representing the bulk of previous research efforts [29]. Many studies have investigated the relationship between various genes or metabolic pathways and cognitive impairment [30]. Two such putative pathways include folate metabolism in the form of homocysteine levels, and lipid metabolism in the form of the *ApOE4* gene [27, 28, 30]. 

Compelling evidence has identified elevated homocysteine levels as a marker for cardiovascular disease, stroke, vascular dementia, and Alzheimer’s disease in the elderly [31–37], as well as for poorer outcomes from traumatic brain injury [38], stroke, and dementia. Elevated homocysteine levels have also been associated with histopathologically confirmed Alzheimer’s disease, [34] as well as hippocampal atrophy and white matter hyperintensity and volume loss [39, 40]. The pathophysiology underlying this association is complex and incompletely understood, but potential mechanisms for ND likely include impaired cerebrovascular circulation, inflammation, and endothelial dysfunction [41, 42]. To our knowledge, ours is the first study to evaluate homocysteine levels in the context of radiotherapy, but there is sufficient biological plausibility to implicate that elevated serum homocysteine in the pathogenesis of radiation-induced ND. The significance of this finding lies in the ability to lower homocysteine levels with readily-available vitamin supplements [43]. 

It is important to note that there are differences between labs in what is considered a “normal range” for homocysteine. Homocysteine level is a continuous variable that can be converted to a categorical value by defining a cut-off for what is considered “elevated”. Logistic regression revealed highly significant relations to MMSE, MRI, Patient, and Physician Assessment when the level was dichotomized to “normal vs high”, as described above. For example, 41 patients (MMSE > 24 group) had levels measured at a lab with a normal range of 4.5 to 12.4, and in the MMSE < 24 group, nineteen patients had homocysteine levels measured at a lab with normal range of 5 to 15 and the remaining twenty patients measured at as many as seven different labs.

Several methods were used for examining groups with homocysteine as a continuous variable (separately, combined, and combined with recalibration) and all methods produced significant and highly consistent results. The most significant results arose from combining groups without recalibrating. We believe the most significant finding of this analysis is the need to standardize and use homocysteine levels as a continuous variable. The categorical cutoff of above/within lab normal is arbitrary, and as demonstrated in Supplemental Fig. 1, may offer good predictive value for high levels, but not as good for low levels. The goodness of fit for the logistic regression on homocysteine indicates that knowing the exact and standardized value of homocysteine can be of benefit to a physician guiding patients through these difficult medical treatments. This is reflected in our online nomogram where homocysteine is measured as a continuous variable for outcome prediction.

*ApOE4* is also a well-established risk factor for dementia, especially after brain insults [44]. The *ApOE* gene encodes for apolipoprotein E, a normal constituent of many lipoprotein particles which acts as a ligand for membrane receptors mediating lipoprotein uptake [45]. The gene has three common alleles: ε2, ε3, and ε4, with the ε4 variant representing the primary genetic risk factor for Alzheimer’s disease. The role of this allele in the pathogenesis of Alzheimer’s disease involves multiple pathways including lipid transport and metabolism [46], as well as neuronal repair [47], and confers a 60% lifetime risk of Alzheimer’s dementia by age 85 in homozygotes [48]. Most^39,40^ but not all^41^ studies suggest that the *ApOE4* genotype increases the risk of neurocognitive dysfunction in patients with brain metastasis undergoing WBRT [14, 49]. The *ApOE4* allele is also associated with cardiovascular disease [50, 51], and a recent, large, population-based study estimated that one-fourth of the *ApOE4-*mediated effects on cognition were caused by cerebral microbleeds [52]. Taken together, these findings suggest a potential synergistic relationship between *ApOE4* and elevated homocysteine resulting in vascular dysfunction and injury leading to dementia, and again provide biological plausibility to our finding that these two variables are strongly associated with radiation-induced ND.

Recently, Tohidinezhad and colleagues [34] developed a predictive model for ND incorporating age, weight, chemotherapy treatment, and radiation dose to various brain structures to predict ND. However, some of the dosimetry calculations are complex, and must occur *after* radiation is delivered since they require knowledge of the radiation dosage and technique. Our approach is unique in that only factors accessible *before* the start of radiotherapy are required to calculate a quantitative estimate of ND risk. A few other recent studies have evaluated the effect of *ApOE* genotype and serum levels of associated proteins with radiation [14, 53] and chemotherapy-induced [54] neurocognitive decline. Huntoon et al. (2023) retrospectively evaluated biomarkers associated with cognitive decline after radiotherapy for brain metastasis and found no differences between *ApOE* genotypes but found an association with actual serum levels of ApoE proteins [55]. Importantly, some of these studies [53, 55] were restricted to patients with brain metastases, and most received whole brain irradiation. Over half of the patients in our study had primary brain tumors, and only about one-third received WBRT (the rest were treated with IMRT). Our nomogram is therefore more broadly generalizable to a wider breadth of patients and treatment modalities.

In addition to facilitating discussions of treatment-associated risk, the use of our predictive algorithm could also facilitate the study of preventive interventions in high-risk populations. In fact, although the results of our study must not be interpreted to suggest a causal relationship between homocysteine level or the *ApOE4* allele and radiation-associated central nervous system toxicity, both of these risk factors are potentially remediable. As a mediator of lipoprotein uptake, the adverse effects of *ApOE* may be mitigated by supplementation with choline [56] or statin medications [57], the adverse effects of elevated homocysteine could possibly be ameliorated by antiplatelet or anticoagulant medications to avoid thromboembolic events [58], and B vitamin supplementation to lower homocysteine levels [59]. The potential of these interventions warrants evaluation in prospective studies.

Several important limitations of this study require emphasis. This was a retrospective analysis, and some potentially important information, including radiation dosimetry to different brain structures (for example, the temporal lobes, hippocampus, and pituitary), and concurrent use of statin and antiplatelet therapy were not collected. Lab heterogeneity in homocysteine values, as described above, should again be acknowledged. Also, no patients in this study received memantine or hippocampal avoidance treatment plans. We did not include patients who received stereotactic radiosurgery, but ND remains a problem in this population as well, and warrants separate evaluation. The size of our patient cohort is small, and confined to a single institution. Because of this small size, dividing our patient sample into training and validation cohorts was not feasible. Our nomogram therefore requires validation in larger and more diverse patient populations. Although we believe that the outcome measures used in this study are reliable and clinically meaningful [60–63], the MMSE is primarily a screening tool. There are now newer, more robust methods for evaluating ND and may provide a more comprehensive evaluation. Therefore, the risk factors identified in our study need to be studied further using additional endpoints [53–55].

## Conclusion

Radiation-induced neurocognitive decline is a debilitating late effect of cranial irradiation, which is increasing in frequency as a consequence of improved survival for patients with primary and metastatic brain tumors. We have identified two novel risk factors for ND – elevated homocysteine and *ApOE* genotype – from a cohort of intensively studied patients with brain tumors. Combining elevated homocysteine level and the presence of an *ApOE4* allele with age *≥* 65 (a previously well-described risk factor), we have created a very user-friendly interactive nomogram for prediction of ND risk prior to the start of radiotherapy. The accuracy of this predictive nomogram is being evaluated in larger prosecutive studies using additional outcome measures for radiation-induced cognitive dysfunction.

## Supplementary Information

Below is the link to the electronic supplementary material.

Supplementary Material 1

Supplementary Material 2

Supplementary Material 3

## Data Availability

The data will be made available upon reasonable request.

## Declarations

### Previous presentation

Portions of this study were presented at the 2022 American Society of Clinical Oncology Annual Meeting, Chicago, IL.
