# Supplementary material for: Predicting late radiation-associated neurocognitive and endocrine toxicity in patients with brain tumors
Source: J Neurooncol. 2026 May 28;178(1):26. doi: 10.1007/s11060-026-05646-9 (PMC13219158; doi:10.1007/s11060-026-05646-9)
Supplement: Supplementary file 1 — Supplementary Material 1 [file 11060_2026_5646_MOESM1_ESM.docx]

Supplemental Table 1. Frequency of Outcomes used for nomogram prediction

| Outcome Measure | Frequency of Occurrence | | |
| --- | --- | --- | --- |
|  | **Study Cohort** | **Literature** | **References** |
| MMSE < 24 | 39/80 (48.75%) | 11% [6-19]/15%, 12% | Taylor (1998)[1], Corn (2008)[2] |
| Endocrine Dysfunction | 13/80 (16.25%) | >43%, ~20%, >25% | Kyriakakis (2016, 2019)[3], [4], Taku (2017)[5], Chapon (2025)[6] |
| White Matter Change on MRI | 34/80 (42.5%) | 50%, 35%, 8% | Stokes (2015)[7], Sabsevitz (2013)[8], Corn (1994)[9] |
| Physician Assessment of Cognitive Status | 36/80 (45.0%) | 28%, up to 90%, 50-90% | Crossen (1994)[10], Greene-Schloesser (2013) [11], [12], Makale (2017) |
| Patient Assessment of Cognitive Status | 46/80 (57.5%) | 30%, ~63% | Zeng (2025)[13], Gondi (2023)[14] |

References

[1] B. V Taylor *et al.*, “Effects of radiation and chemotherapy on cognitive function in patients with high-grade glioma.,” *Journal of Clinical Oncology*, vol. 16, no. 6, pp. 2195–2201, Jun. 1998, doi: 10.1200/JCO.1998.16.6.2195.

[2] B. W. Corn *et al.*, “Prospective Evaluation of Quality of Life and Neurocognitive Effects in Patients With Multiple Brain Metastases Receiving Whole-Brain Radiotherapy With or Without Thalidomide on Radiation Therapy Oncology Group (RTOG) Trial 0118,” *International Journal of Radiation Oncology*Biology*Physics*, vol. 71, no. 1, pp. 71–78, 2008, doi: https://doi.org/10.1016/j.ijrobp.2007.09.015.

[3] N. Kyriakakis *et al.*, “Hypothalamic-pituitary axis irradiation dose thresholds for the development of hypopituitarism in adult-onset gliomas,” *Clin. Endocrinol. (Oxf).*, vol. 91, no. 1, pp. 131–140, Jul. 2019, doi: https://doi.org/10.1111/cen.13971.

[4] N. Kyriakakis *et al.*, “Pituitary dysfunction following cranial radiotherapy for adult-onset nonpituitary brain tumours,” *Clin. Endocrinol. (Oxf).*, vol. 84, no. 3, pp. 372–379, Mar. 2016, doi: https://doi.org/10.1111/cen.12969.

[5] N. Taku, M. Gurnell, N. Burnet, and R. Jena, “Time Dependence of Radiation-induced Hypothalamic–Pituitary Axis Dysfunction in Adults Treated for Non-pituitary, Intracranial Neoplasms,” *Clin. Oncol.*, vol. 29, no. 1, pp. 34–41, 2017, doi: https://doi.org/10.1016/j.clon.2016.09.012.

[6] J. Chapon *et al.*, “Pituitary dysfunction after cranial radiotherapy for brain tumor,” *Ann. Endocrinol. (Paris).*, vol. 86, no. 4, p. 101722, 2025, doi: https://doi.org/10.1016/j.ando.2025.101722.

[7] T. B. Stokes *et al.*, “White matter changes in breast cancer brain metastases patients who undergo radiosurgery alone compared to whole brain radiation therapy plus radiosurgery,” *J. Neurooncol.*, vol. 121, no. 3, pp. 583–590, 2015, doi: 10.1007/s11060-014-1670-4.

[8] D. S. Sabsevitz *et al.*, “The role of pre-treatment white matter abnormalities in developing white matter changes following whole brain radiation: a volumetric study,” *J. Neurooncol.*, vol. 114, no. 3, pp. 291–297, 2013, doi: 10.1007/s11060-013-1181-8.

[9] B. W. Corn *et al.*, “White matter changes are correlated significantly with radiation dose. Observations from a randomized dose-escalation trial for malignant glioma (radiation therapy oncology group 83-02),” *Cancer*, vol. 74, no. 10, pp. 2828–2835, Nov. 1994, doi: 10.1002/1097-0142(19941115)74:10<2828::AID-CNCR2820741014>3.0.CO;2-K.

[10] J. R. Crossen, D. Garwood, E. Glatstein, and E. A. Neuwelt, “Neurobehavioral sequelae of cranial irradiation in adults: a review of radiation-induced encephalopathy.,” *Journal of Clinical Oncology*, vol. 12, no. 3, pp. 627–642, Mar. 1994, doi: 10.1200/JCO.1994.12.3.627.

[11] D. Greene-Schloesser, E. Moore, and M. E. Robbins, “Molecular pathways: radiation-induced cognitive impairment.,” *Clin. Cancer Res.*, vol. 19, no. 9, pp. 2294–2300, May 2013, doi: 10.1158/1078-0432.CCR-11-2903.

[12] M. T. Makale, C. R. McDonald, J. A. Hattangadi-Gluth, and S. Kesari, “Mechanisms of radiotherapy-associated cognitive disability in patients with brain  tumours.,” *Nat. Rev. Neurol.*, vol. 13, no. 1, pp. 52–64, Jan. 2017, doi: 10.1038/nrneurol.2016.185.

[13] H. Zeng *et al.*, “Impact of HA-PCI on self-reported cognitive functioning and brain metastases in small-cell lung cancer: Pooled findings of NCT01780675 and PREMER trials,” *Lung Cancer*, vol. 199, p. 108036, 2025, doi: https://doi.org/10.1016/j.lungcan.2024.108036.

[14] V. Gondi *et al.*, “Sustained Preservation of Cognition and Prevention of Patient-Reported Symptoms With Hippocampal Avoidance During Whole-Brain Radiation Therapy for Brain Metastases: Final Results of NRG Oncology CC001,” *International Journal of Radiation Oncology*Biology*Physics*, vol. 117, no. 3, pp. 571–580, 2023, doi: https://doi.org/10.1016/j.ijrobp.2023.04.030.
