# Supplementary material for: Predicting late radiation-associated neurocognitive and endocrine toxicity in patients with brain tumors
Source: J Neurooncol. 2026 May 28;178(1):26. doi: 10.1007/s11060-026-05646-9 (PMC13219158; doi:10.1007/s11060-026-05646-9)
Supplement: Supplementary file 2 — Supplementary Material 2 [file 11060_2026_5646_MOESM2_ESM.doc]

Supplemental Figure 1: Association between individual Homocysteine levels and different neurocognitive outcomes
